# Do environmental stimuli modify sensitive plant (*Mimosa pudica* L.) risk assessment?

**DOI:** 10.1371/journal.pone.0294971

**Published:** 2023-12-21

**Authors:** Charlotte M. Cosca, Justin A. Haggard, Halli M. Kato, Eleni M. Sklavenitis, Daniel T. Blumstein

**Affiliations:** Department of Ecology and Evolutionary Biology, University of California Los Angeles, Los Angeles, California, United States of America; Indian Institute of Science, INDIA

## Abstract

Although plants and animals both assess their environment and respond to stimuli, this reaction is considered a behavior in animals and a response in plants. Responses in plants are seen within various timescales- from the nanosecond stimuli is presented to a lifelong progression. Within this study, we bridge the gap between animal behavioral studies and plant response. Sensitive plants (*Mimosa pudica* L.) are an ideal subject for this due to the rapid closure of their primary leaflets when touched. We designed a multimodal, or stress combination, experiment to test two hypotheses with sensitive plants: if they could be distracted and if they would alter their risk assessment when exposed to external stimuli (wind and sound). To evaluate the distraction hypothesis, we measured an individual’s latency to close, hypothesizing that if the plants were distracted, they would take longer to close. To evaluate the uncertain risk hypothesis, we quantified the latency to reopen, hypothesizing that if the plants were uncertain, they would take longer to reopen. We also quantified the number of pinnae closed on the selected stem to test for changes in risk assessment across treatments. We expected the unimodal treatments would distract or alter risk assessment, and the multimodal treatment would elicit an enhanced response. Multimodal stimuli had a significant effect on the number of pinnae closed before the tap, but we found no evidence that plants were distracted by any stimulus tested. We found that temperature had a significant effect on the latency to close, and that plants modified their risk assessment when exposed to experimental wind stimuli. By manipulating environmental stimuli, we found that sensitive plants trade-off energy and perceived risk much in the way that is commonly found in animals. Framing the study of plants’ responses to environmental stimuli as behavioral questions may generate new insights.

## Introduction

Animals and plants both assess risks in their environment and react to stimuli. While that is generally accepted as a behavioral response for animals, it is more controversial in the case of plants. We explore how plants process and rapidly respond to their environment, which in animals might be a measure of their cognitive abilities [1,2]. Plants respond to a variety of external stimuli by altering their root or stem path with regards to water, light, gravity and the avoidance of salt [3–7]. By doing so, they increase their access to favorable growing conditions and can correct growth patterns. In response to herbivory, *Acacia sieberiana* grows longer spines, decreases leaf size, and increases hydrogen cyanide concentration throughout the plant [8,9]. In contrast to animals, plants have adapted to their environment by developing responses to stimuli over various time scales.

Some plants have rapid responses to environmental stimuli. These rapid responses indicate the adaptiveness of the reaction to the organism [10]. For instance, when the plant *Arabidopsis thaliana* L. detects the vibration of a caterpillar (*Pieris rapae*) chewing, it increases production of toxic aliphatic glucosinolates which protects them from herbivory [11]. Some species within the genus *Passiflora* increase pollen collection by moving their androgynophore toward pollinators as they feed on the flower’s nectar [12]. *Mimulus guttatus* prevents self-pollination by closing its stigmas for a period after a pollinator’s visit [13]. At an even shorter time scale researchers have analyzed plants’ cell-to-cell signaling (with reactive oxygen species, calcium ions, and electrical molecular components) immediately after local stimulation [14]. These rapid response times are more easily observed than those on large time scales and help provide a basis for applying methods typically used to study animal behavior to plants.

Sensitive plants (*Mimosa pudica* L.) are a model organism to study plant responses because they have a rapid antipredator response [15]. When touched, they close (within ca. 15 s) their primary leaflets and remain closed for 5–15 min [16]. Hagihara et al. [17] recently confirmed the long-standing assumption that this closure repels predators [16,18]. While pinnae are closed photosynthetic rates are reduced up to 40% [16]. This creates a trade-off between acquiring energy via photosynthesis and resistance to herbivory, which is enhanced during low light [16,19–21]. Similar to animals, this trade-off within sensitive plants is modified as the leaf ages and will habituate to non-damaging stimuli [22,23]. Recent studies have found that free-living sensitive plants can distinguish and remember multiple stimuli, do not habituate to harmful stimuli, and will habituate differently based on age and distance from inflorescence [22,24].

Organisms are limited in their ability to allocate attention to assessment because attention is finite [25]. Attention is a controversial term within plant ecology; we define plant attention as “an overall level of alertness or ability to engage with surroundings” [26]. By focusing too much attention on risk assessment, individuals may limit their ability to acquire resources [27]. For instance, mammalian prey of rat snakes (*Elaphe climacophora*) are distracted by the snake’s rapid tail movements which mimic prey [28]. Hermit crabs (*Coenobita clypeatus*) have an impaired ability to respond to predator cues when exposed to other auditory and visual stimuli [25]. Sensitive plants may also become distracted as more environmental stimuli are present and this would be seen through slower closing times (i.e., latency to close). We define distraction regarding plants as an impaired ability to respond or decreased awareness of a true threat [26].

All organisms are capable of making risk-sensitive decisions under uncertainty [21,29–32]. These decisions trade-off between responding to a predatory cue when a predator is not present (false-positive) and failing to respond to a predator cue when a predator is present (false-negative). Error management theory (EMT) explains how the cost of false-positive and false-negative outcomes are weighed while in a state of uncertainty [33]. In theory, allocating more time and energy to risk assessment could reduce uncertainty, but this too is costly if it prevents individuals from engaging in behaviors that increase their fitness (i.e., foraging, courting, mating, etc.). Uncertainty within sensitive plants can be measured through increased times they remain closed (i.e., hiding time).

We studied free-living sensitive plants to investigate if plants could be distracted by environmental stimuli and whether exposure to environmental stimuli altered risk assessment. We experimentally created wind and noise because plants are regularly exposed to both and, in principle, both could affect attentional processes and risk assessment as seen in animals [25,34,35]. Wind could distract an individual or increase uncertainty about whether the plant was being touched. Sound could have a similar effect as a new vibration, propagating as an audible wave, that the plant must process and potentially respond to [36]. Although white noise has not been sufficiently tested within the plant community, within the animal community, it can lead to enhanced responsiveness due to its novelty to the organism [35,37,38].

We designed a multi-modal, or stress combination, experiment [39–44] to test sensitive plant threat assessment for environmental stimuli. The multimodal phenomenon occurs when stimuli provide information in more than one sensory modality simultaneously. Plants are constantly being presented with multimodal stimuli within their environment, thus studying plants’ reactions to multimodal treatments provides a foundation to study the function and evolution of perception [45,46]. We used portable fans to generate wind across the leaves and a portable speaker to broadcast white noise. We expected that either stimuli in isolation could potentially distract individuals or modify their risk assessment. If plants, like many animals, perceptually bind stimuli, we expected a multimodal treatment would lead to an enhanced response compared to unimodal treatments [39,41].

## Methods

**Fig 1:**
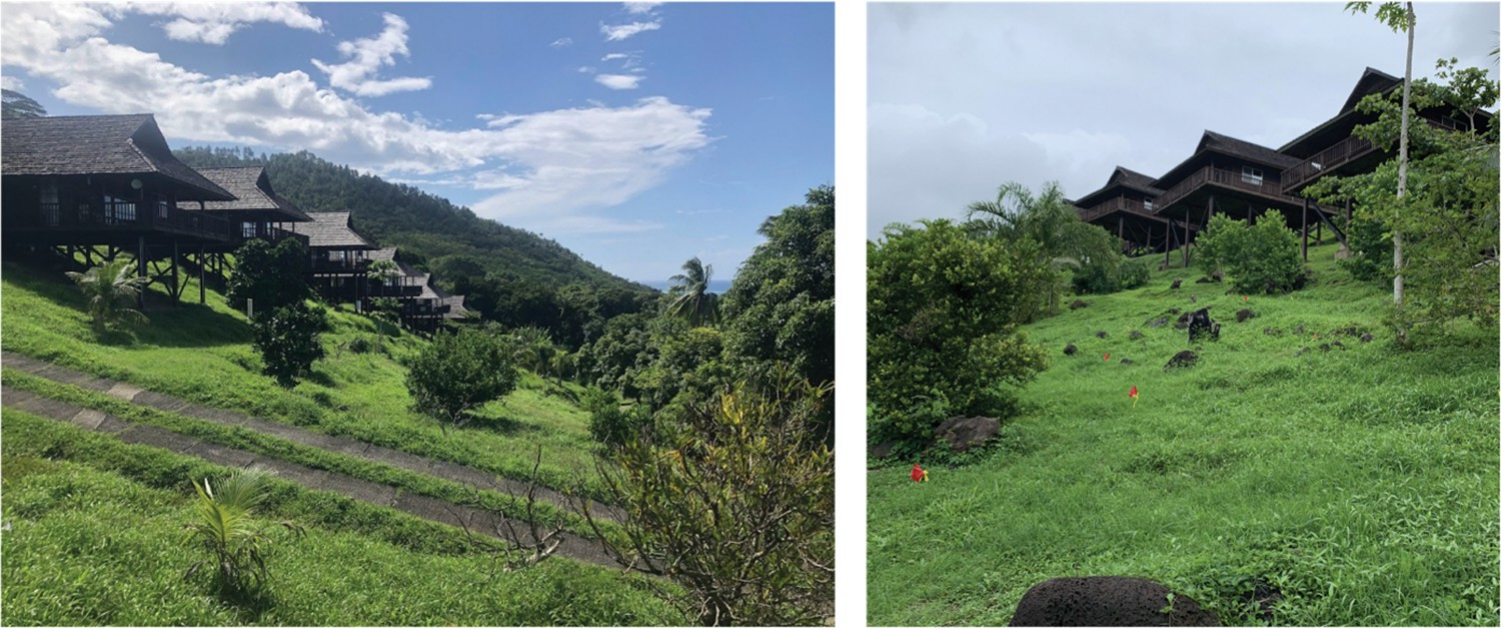
Photos of study site at the Gump Marine Lab, Moorea, French Polynesia.

**Fig 2:**
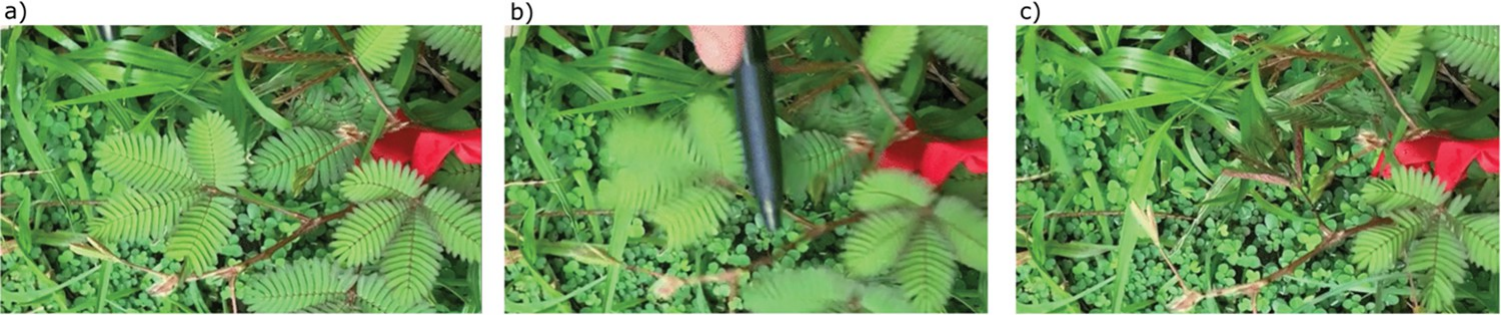
Photos of experimental tap. a) Before experimental tap, b) Experimental tap on the petiole, c) After experimental tap. Also pictured is the tag below the third fully developed leaf from the apex towards the basis.

We studied sensitive plants at the UC Berkeley Gump South Pacific Research Station (17°29’29.6"S 149°49’42.7"W) in Moorea, French Polynesia between 14 January and 8 February 2022. Research was conducted with protocols issued on 19 November 2021 by the Government of French Polynesia. A total of 32 plants were studied on a NE facing hillside below unoccupied bungalows, in an area with direct sunlight (Fig 1). Subjects were ≥ 5 m away from each other. All subjects were tagged below the second leaf from the apex towards the basis (Fig 2). As leaf age can affect closure rates [22], we performed all treatments on the second, fully developed leaf near the apex on the chosen branch and continued on this leaf as the plant grew (Fig 2). For initial measurements, we recorded the number of pinnae on the chosen leaf, the number of leaves on the subject, the height of the subject, and the slope under the subject. Trials began 48 h after initial measurements were taken, and at least 48 h after the prior trial. Due to overgrowth by other species, we cut the surrounding plants between treatment days two and three to better isolate our test subjects.

### Experiment design

We conducted a multi-modal experiment where individual plants received four different treatments in a Latin Square design: *a*. control, *b*. wind, *c*. white noise, and *d*. simultaneous wind and white noise (Table 1). The multimodal treatment was simultaneous presentation of wind and white noise. First, the treatment and video would start. After one minute, an experimenter would ‘tap’ the individual plant on the chosen leaf’s petiole with a pen (Paper Mate Ballpoint 300 RT, Newell Office Brands, India) to trigger leaf closure (Fig 2). The tap was performed by a single experimenter to ensure reproducibility and was practiced beforehand on nonexperimental plants. The treatment and video would continue until all pinnae were completely open. A trial was only conducted when the Beaufort scale was ≤ 2 and it was not raining. We aimed to perform each set of treatments every 48 h to decrease the chances of habituation and allow the individual time to recover. However, we had to terminate many experiments due to increases in wind and the onset of rain as these are other forms of mechanical stimuli that interfered with our ability to properly measure the latency to reopen. We terminated any trial where increased wind or rain activity closed any pinnae before all pinnae opened completely. After completing each set of planned treatments, we re-conducted any terminated trials at least 48 h after prior trials (Table 1).

**Table 1:** Latin-square design is shown on days 1–4. Terminated trials are shown crossed out. Reconducted trials are shown on days 5–7.

Plant Number	Day 1	Day 2	Day 3	Day 4	Day 5	Day 6	Day 7
1	Control	Wind	Sound	Wind + Sound	Control		
2	Wind + Sound	Control	Wind	Sound	Wind + Sound		
3	Sound	Wind + Sound	Control	Wind	Sound		
4	Wind	Sound	Wind + Sound	Control			Wind
5	Control	Wind	Sound	Wind + Sound	Control	Wind	Sound
6	Wind + Sound	Control	Wind	Sound			Wind
7	Sound	Wind + Sound	Control	Wind			Sound
8	Wind	Sound	Wind + Sound	Control			
9	Control	Wind	Sound	Wind + Sound	Wind + Sound		
10	Wind + Sound	Control	Wind	Sound			
11	Sound	Wind + Sound	Control	Wind	Sound		
12	Wind	Sound	Wind + Sound	Control			Wind + Sound
13	Control	Wind	Sound	Wind + Sound			Wind
14	Wind + Sound	Control	Wind	Sound	Sound	Wind	
15	Sound	Wind + Sound	Control	Wind		Control	Wind + Sound
16	Wind	Sound	Wind + Sound	Control	Wind		
17	Control	Wind	Sound	Wind + Sound	Control	Wind	
18	Wind + Sound	Control	Wind	Sound	Wind + Sound		
19	Sound	Wind + Sound	Control	Wind	Control		
20	Wind	Sound	Wind + Sound	Control			
21	Control	Wind	Sound	Wind + Sound			
22	Wind + Sound	Control	Wind	Sound			
23	Sound	Wind + Sound	Control	Wind		Sound	Sound
24	Wind	Sound	Wind + Sound	Control	Wind + Sound		
25	Control	Wind	Sound	Wind + Sound	Wind		Wind + Sound
26	Wind + Sound	Control	Wind	Sound	Wind + Sound	Wind	
27	Sound	Wind + Sound	Control	Wind	Wind + Sound	Control	Control
28	Wind	Sound	Wind + Sound	Control	Sound		Control
29	Control	Wind	Sound	Wind + Sound	Control		
30	Wind + Sound	Control	Wind	Sound			Sound
31	Sound	Wind + Sound	Control	Wind			
32	Wind	Sound	Wind + Sound	Control			

We conducted experiments between 7:00 and 16:30. The average photoperiod range during the time of our experiment was 5:44 to 18:38. Before each treatment, we recorded the date, time, treatment, observer, start time, Beaufort scale, cloud coverage (Oktas), ambient noise in dB with slow time-weighting and A-weighting (NIOSH sound level meter National Institute for Occupational Safety and Health, Version 1.2.5.63) and temperature (°C with the Amprobe IR-712 12:1 IR, 2013 Amprobe Test Tools, China) (Table 2). We began experiments when all pinnae were open. We recorded experiments using iPhones (Apple Inc. Cupertino, California, iPhone 8+, iPhone 8, iPhone XS).

**Table 2:** Daily averages of temperature, wind, background noise, and cloud cover before each treatment. Cloud cover was not recorded in Oktas during the first two days of the experiment.

Days	Temperature (°C)	Wind (Beaufort)	Noise (dB)	Cloud Cover (Oktas)
1	26.5	1.4	49	
2	26	1.5	48.1	
3	28.4	1.5	48.2	4.9
4	29	0.9	48	0.9
5	26.8	1.3	45.5	6
6	28.5	1.8	41.4	2.3
7	30.6	0.6	43.8	1.8

We simulated wind using a portable fan (1.56 m/s, Snawowo, Model # 5978S2Q, Longgang District, Shenzhen), placed 30 cm from the subject and projected at the ventral side of the leaf. We created a 30 min track of white noise, using Audacity (Audacity® Version 3.1.3), and broadcast it at 78.6 dB (measured 1 m away, tested with slow time-weighting and A-weighting), through a UE Boom 2 Wireless Bluetooth Speaker (UE, 984–000553, China). The average background noise level for the area was 47.5 dB (calculated from measurements taken before each trial). For consistency, the speaker and fan were set up 90° apart.

### Video analysis

We analyzed videos to quantify the timing of plant response with Adobe Premiere Pro (Adobe Inc. Version 22.2). We worked in pairs to score videos, and all four experimenters practiced scoring the videos together to reduce interobserver variability. We quantified latency to close, hiding time, and the total number of pinnae closed before experimental tap. We counted the number of pinnae open before the tap and subtracted that from the total number of pinnae to find the total pinnae closed. We divided that number by the total pinnae closed to find the proportion closed.

We quantified distraction as the latency for the target leaf to fully close after the experimental tap. We measured to the nearest frame and calculated the time in seconds. Closure was defined as the frame in which all pinnae ceased to move and was analyzed independently of stem movement associated with the tap.

We quantified risk assessment as the latency for the target leaf to fully reopen to its initial state after closure. We stopped the video at this point and recorded the number of frames. We then converted the number of frames to time in seconds. If any pinnae remained closed, we defined fully reopened as the time when all but those pinnae opened. Pinnae that were immobile for 3 min would be considered ‘remaining closed’. Additionally, if pinnae interacted with the surrounding plants or insects and remained closed then these pinnae were not used to define the latency to reopen.

### Statistical analysis

For analysis we eliminated trials conducted while it was raining, during strong wind events (Beaufort > 2), high temperatures (≥ 35°C), abnormally large latencies to reopen (> 1,000 s), and when there were technological malfunctions. This left 113 trials in our final data set across 32 individuals. Treatments were evenly distributed among individuals (C, M, W = 28; S = 29). Before fitting models to study distraction and assessment, we checked to see if environmental conditions were confounded by conducting a chi-squared test to see if there was a relationship between Beaufort and treatment type (there was not, *P* = 0.620). To test for potential multicollinearity, we correlated all continuous independent variables; there was none (correlation coefficients all < 0.49). The data for latency to re-open and latency to close were transformed to better fit a normal distribution.

We fitted three sets of linear mixed models–one for each of our dependent variables: latency to open, latency to close, and the number of pinnae closed offset by the total number of pinnae. For each dependent variable, we began with a model that included the following fixed effects: treatment, treatment day (number of trials performed thus far on each individual) to account for any habituation [47], Beaufort scale, and ambient noise (dB) because our stimuli had physical (wind) and acoustic elements. Plant identity (or each subject) was included as a random effect in all models. We then systematically added potential obscuring variables one by one–temperature, plant length, total number of pinnae–to these basic models and included these additional covariates in the final model only if significant.

Models were fitted using the R package “lme4” package [48]. We tested our models to ensure that they met the assumptions of linear mixed models with a Gaussian distribution using the “check model” function included in the “performance” package in R [49]. We used the emmeans package [50] to test for the pairwise difference between treatment types, which we ran with no adjustments for our multiple planned comparisons [see 51–54]. We also used the emmeans package to calculate Cohen’s d–a measure of effect size. We used the partR2 package in R [55] to calculate and compare the marginal and conditional part R^2^ values for the fixed effects.

## Results

### Immediate response to treatment

**Fig 3:**
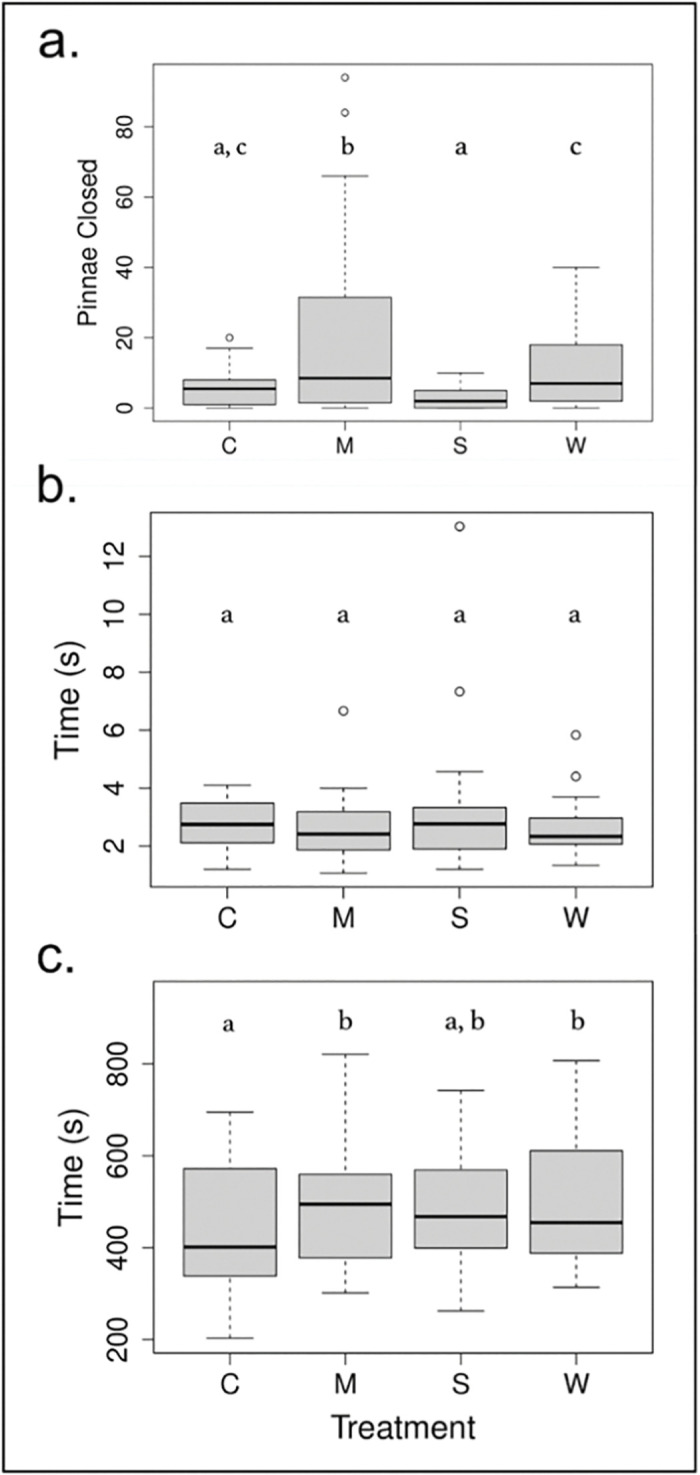
a. Number of pinnae closed, b. latency to open, c. latency to close as a function of treatment. Treatments include C (control), M (multimodal), S (sound), and W (wind).

The number of pinnae closed on the leaf prior to the experimental tap varied significantly with treatment (*P* < 0.001), and the planned comparison of treatments showed that the number of pinnae closed in multimodal treatments was significantly higher from control (*P* < 0.001; d = 1.006 ± 0.281), sound treatment (*P* < 0.001; d = 1.310 ± 0.302), and wind treatment (*P* = 0.013; d = 0.756 ± 0.302; Table 3A; Fig 3A). The other treatments were not significantly different from the control (*P* > 0.05 for C-S and C-W contrasts; Fig 3A). There was no significant effect of treatment days (*P =* 0.944), noise (*P* = 0.755), or Beaufort scale (*P* = 0.586) (Table 3A; Table 4A). Treatment explained 11.2% of the variance in the number of pinnae closed, while plant identity explained 16.7% of the variance in the number of pinnae closed (Table 3A; Table 4B).

**Table 3:** P-values and part R^2^ values for fixed effects in each model. Marginal part R^2^ values quantify the variance explained by each fixed effect, whereas the conditional part R^2^ values quantify the variance explained by both the fixed effect and the random effect (plant number).

	*p* value	Conditional Part R^2^	Marginal Part R^2^
**(a) Pinnae Closed**			
Treatment	**< 0.001**	0.279	0.112
Treatment Days	0.944	0.167	< 0.001
Noise	0.755	0.171	0.004
Beaufort	0.586	0.170	0.003
Conditional R^2^ 0.588		Marginal R^2^ 0.421	
**(b) Latency to Close**			
Treatment	0.417	0.141	0.020
Treatment Days	0.242	0.132	0.011
Noise	0.197	0.134	0.013
Beaufort	0.977	0.122	< 0.001
Temperature	**< 0.001**	0.234	0.113
Conditional R^2^ 0.271		Marginal R^2^ 0.150	
**(c) Latency to Open**			
Treatment	0.073	0.172	0.047
Treatment Days	**0.010**	0.173	0.048
Noise	0.630	0.126	< 0.001
Beaufort	0.059	0.153	0.028
Conditional R^2^ 0.287		Marginal R^2^ 0.162	

**Table 4:** : Estimates for fixed effects in each model; B: Variance and SD of random effects in each model.

A	Est.	SE
**(a) Pinnae Closed**		
Treatment Days	0.078	1.097
Noise	0.104	0.333
Beaufort	-1.388	2.542
**(b) Latency to Close**		
Treatment Days	0.012	0.011
Noise	-0.003	0.003
Beaufort	< -0.001	0.023
Temperature	-0.025	0.006
**(c) Latency to Open**		
Treatment Days	0.019	0.007
Noise	-0.001	0.002
Beaufort	-0.033	0.017
**B**	Variance	Standard Deviation
**(a)** Pinnae Closed		
**Plant ID**	100.8	10.04
**(b)** Latency to Close		
**Plant ID**	0.004	0.061
**(c)** Latency to Open		
**Plant ID**	0.002	0.046

### Test of distraction

Plants did not vary their latency to close following an experimental tap as a function of treatment (*P* = 0.417; Table 3B). There were no significant differences in the latency to close between control and the other treatments, or between the other treatments and each other (Fig 3B), and the effect sizes tended to be larger in the contrasts between different stimulus treatments (d_M-S_ = 0.461 ± 0.288; d_M-W_ = 0.240 ± 0.297; d_S-W_ = 0.221 ± 0.277) than in the contrasts between stimulus treatments and the control (d_C-M_ = 0.307 ± 0.272; d_C-S_ = 0.154 ± 0.292; d_C-W_ = 0.067 ± 0.299) (Fig 3B). There were also no significant effects of treatment days (*P* = 0.242), noise (*P* = 0.197), or Beaufort (*P* = 0.977) on the latency to close (Table 3B; Table 4A). However, we found that at higher temperatures (Est. = -0.025 ± 0.006), plants closed more slowly (*P* < 0.001) (Table 3B; Table 4A). Temperature explained 11.3% of the variance, while plant identity explained 12.1% of the variance in latency to close (Table 3B; Table 4B).

### Test of modified risk assessment

There was a moderately significant effect of treatment on the latency to reopen (*P* = 0.073; Table 3C). Plants varied their latency to reopen significantly between control and wind treatments (*P* = 0.016; d = 0.735 ± 0.303) as well as between control and multimodal treatments (*P* = 0.040; d = 0.567 ± 0.274; Fig 3C). There was no significant difference between control and sound treatments (*P* = 0.113; d = 0.466 ± 0.293), nor between multimodal and wind treatments (*P* = 0.569; d = 0.168 ± 0.295) (Fig 3C). In addition, plants took longer to reopen (Est. = 0.019 ± 0.007) as a function of treatment days (*P* = 0.010) and there was a moderately significant effect of environmental wind speed (*P* = 0.059), but there was no effect of ambient noise (*P* = 0.630; Table 3C; Table 4A). Treatment explained 4.7% of the variance in reopening time, while plant identity explained 12.5% of the variance in reopening time (Table 3C; Table 4B).

## Discussion

Overall, the multimodal experimental presentation of wind and white noise modified sensitive plant response. Plants closed more of their pinnae at the start of the treatment when exposed to the multimodal stimuli. Wind and sound treatments alone did not have a significant effect on the number of pinnae closed when compared to the control, and both these treatments were statistically different from the multimodal treatment. This suggests that the multimodal treatment had an additive effect on the plant’s initial assessment of risk because they produced a greater response when the stimuli were combined rather than when they were introduced independently.

We found no support for the distraction hypothesis because there were no differences in the latency to close as a function of treatment. It is important to note that our experimental wind was 1.56 m/s, which is approximately a 2 on the Beaufort scale. Because we performed experiments when natural wind events occurred at the same Beaufort scale value, it is possible that the experimental wind used was not sufficiently high to distract individuals, but we selected this velocity because it did not result in the plants closing all their pinnae before the experimental tap. Future studies could investigate whether stronger wind distracts sensitive plants. Not unexpectedly, we found significant effects of temperature on the latency to close. It has long been known that temperature may alter closing and opening patterns of this species [56]. What was not known was the relative importance of temperature’s effect; temperature alone explained 11.3% of the variance in closing time.

Our multimodal experimental design allowed us to isolate the effect of wind on the latency to reopen and permitted us to infer that the physical manipulation of plants by the wind modified risk assessment. Wind alone had a large effect (d_C-W_ = 0.735 ± 0.303) and the addition of sound reduced this effect (d_C-M_ = 0.567 ± 0.274). The novelty of white noise may have led to enhanced responsiveness leading to the decreased latency to reopen for the multimodal treatment [35]. However, it would be premature to exclude the possibility that there is an interactive effect with sound given that the number of pinnae closed before we tapped the plant was greatest when both stimuli were combined. Wind alone physically moves the plant’s leaves, which may impede their ability to reopen in ways that sound does not. It is possible that plants may be responding to the mechanical movement as a direct threat, or wind may have impaired the plant’s ability to assess risk due to increased uncertainty. We studied plants during the rainy season and observed that wind was generally associated with changes in cloud cover or rain. Other studies have shown that changes in light availability [47] and temperature [56] modify sensitive plants’ reopening times and the open leaf angle, respectively. Additionally, rain acts as a physical stimulus, triggering leaf closure. These sensitive plant environmental stimuli responses might explain why wind triggers the plants to modify their risk assessment.

While we did not detect a significant effect of sound on the latency to reopen, the effect size compared to the control was moderate (d = 0.466 ± 0.293). This suggests that in a larger sample size we may have detected a significant difference. Previous studies have shown that plants alter their response in the presence of sounds [e.g., 5,11,57,58]. Although studies within the animal kingdom have shown that animals alter their response to white noise [35,38], the previous plant studies used biologically relevant sounds (e.g., herbivores eating a leaf, pollinators flying). Therefore, it is possible that using a more relevant sound in our study may elicit different responses as well.

Unlike what was reported in previous sensitive plant studies [22,24,47,59], we found that plants sensitized rather than habituated to repeated trials. Plants took longer to reopen as the number of trials performed on the individual increased. However, these previous studies were not presenting potentially aversive stimuli to the plants. Our experimental sound was loud and synthetically created, and our experimental wind was moderate yet consistent. Both of these stimuli are different from what sensitive plants would naturally encounter which may have led to the increased sensitivity to the stimuli that was observed. Although we did not test for motor fatigue [60], we do not believe our experiment led to fatigue as each plant had only one treatment every other day. Other sensitive plant studies found that a similar non-harmful stimulus did not cause fatigue within a shorter time interval [22,24]. As our plants were free-living, their responses could have been altered or fatigued by numerous environmental factors before their treatment (for an individual) or during a given day (for the group) [61].

We found that individuality explained over 12% of the variance in each experiment (16.7%—pinnae closed, 12.1%- latency to close, 12.5%—reopening time). Individuality of our subjects could come from differences in morphology or anatomy, behavior due to life cycle stages, developmental noise, or the surrounding environment (e.g., insect exposure, soil composition, the last wind or rain event, etc.) [20,22,47,53,62–64]. Observation of plant individuality has often been disregarded as an experimental error or oversimplified through statistics as population responses [62,65]. Although we were able to quantify individuality, we cannot further specify what caused it despite our analysis of various environmental, anatomical, and geographic factors. Further research in a controlled environment should clarify the cause of this found individuality within sensitive plants.

Our study illustrates the successful application of models of antipredator behavior to investigate plant responses to environmental stimuli. Using sensitive plants, prior studies have evaluated other behavioral concepts that are typically restricted to animals [individuality– 47; habituation- 59, 47; movement-based defense– 17, 18; risk assessment– 22,24]. Trade-offs are a common theme: multiple papers [16,19,20] found sensitive plants traded off photosynthesis and predation risk by showing that individuals closed for a longer period when light was more intense. Sensitive plants allow investigations into plants’ learning and memory as non-neural organisms, bettering our understanding of how different species use information from their environment to survive [23]. Framing the study of how plants respond to environmental stimuli as behavioral questions can be a profitable way to generate new insights.
